# Cytotoxic effects of the cigarette smoke extract of heated tobacco products on human oral squamous cell carcinoma: the role of reactive oxygen species and CaMKK2

**DOI:** 10.1186/s12576-024-00928-1

**Published:** 2024-06-25

**Authors:** Nagao Kagemichi, Masanari Umemura, Soichiro Ishikawa, Yu Iida, Shota Takayasu, Akane Nagasako, Rina Nakakaji, Taisuke Akimoto, Makoto Ohtake, Takahiro Horinouchi, Tetsuya Yamamoto, Yoshihiro Ishikawa

**Affiliations:** 1 Cardiovascular Research Institute, Yokohama City University Graduate School of Medicine, Yokohama, Kanagawa Japan; 2 Neurosurgery, Yokohama City University Graduate School of Medicine, Yokohama, Kanagawa Japan; 3 Oral and Maxillofacial Surgery, Yokohama City University Graduate School of Medicine, Yokohama, Kanagawa Japan; 4 Cellular Pharmacology, Hokkaido University Graduate School of Medicine, Sapporo, Hokkaido Japan

**Keywords:** Heated tobacco products (HTPs), Calcium, Oral cancer, Calcium/calmodulin-dependent protein kinase kinase 2 (CaMKK2), Reactive oxygen species (ROS)

## Abstract

**Background:**

The increasing prevalence of heated tobacco products (HTPs) has heightened concerns regarding their potential health risks. Previous studies have demonstrated the toxicity of cigarette smoke extract (CSE) from traditional tobacco’s mainstream smoke, even after the removal of nicotine and tar. Our study aimed to investigate the cytotoxicity of CSE derived from HTPs and traditional tobacco, with a particular focus on the role of reactive oxygen species (ROS) and intracellular Ca^2+^.

**Methods:**

A human oral squamous cell carcinoma (OSCC) cell line, HSC-3 was utilized. To prepare CSE, aerosols from HTPs (IQOS) and traditional tobacco products (1R6F reference cigarette) were collected into cell culture media. A cell viability assay, apoptosis assay, western blotting, and Fluo-4 assay were conducted. Changes in ROS levels were measured using electron spin resonance spectroscopy and the high-sensitivity 2ʹ,7ʹ-dichlorofluorescein diacetate assay. We performed a knockdown of calcium/calmodulin-dependent protein kinase kinase 2 (CaMKK2) by shRNA lentivirus in OSCC cells.

**Results:**

CSE from both HTPs and traditional tobacco exhibited cytotoxic effects in OSCC cells. Exposure to CSE from both sources led to an increase in intracellular Ca^2+^ concentration and induced p38 phosphorylation. Additionally, these extracts prompted cell apoptosis and heightened ROS levels. *N*-acetylcysteine (NAC) mitigated the cytotoxic effects and p38 phosphorylation. Furthermore, the knockdown of CaMKK2 in HSC-3 cells reduced cytotoxicity, ROS production, and p38 phosphorylation in response to CSE.

**Conclusion:**

Our findings suggest that the CSE from both HTPs and traditional tobacco induce cytotoxicity. This toxicity is mediated by ROS, which are regulated through Ca^2+^ signaling and CaMKK2 pathways.

**Graphical Abstract:**

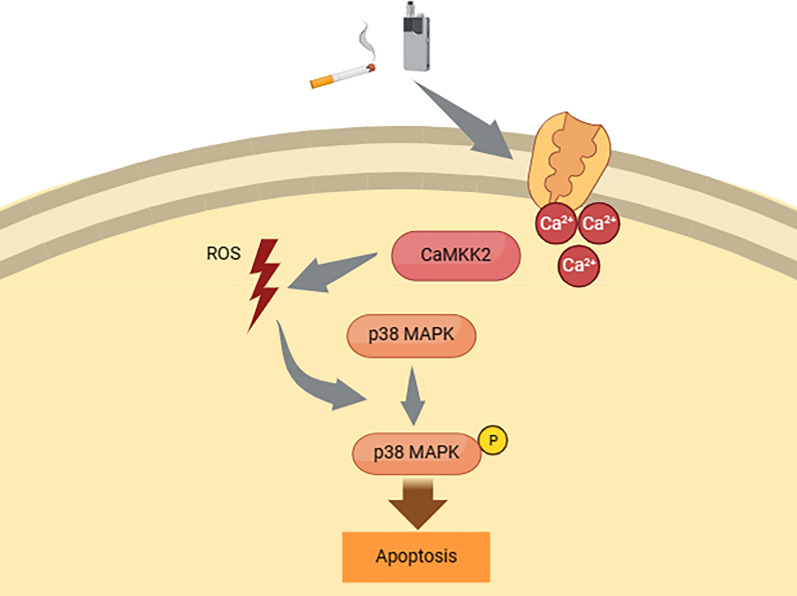

**Supplementary Information:**

The online version contains supplementary material available at 10.1186/s12576-024-00928-1.

## Background

Tobacco is responsible for over 8 million deaths annually worldwide, with over 8 million deaths attributed to direct tobacco use [1]. Tobacco smoke, a blend of over 5000 harmful and carcinogenic chemicals, includes 98 particularly dangerous substances [2]. Smokers have a fivefold higher risk of oral cancer [3].

Cigarette smoke is composed of two phases: the particulate phase and the gas phase. The particulate phase mainly consists of nicotine, tar, and water, while the gas phase contains a number of carbonyl compounds, such as formaldehyde, acrolein, and tobacco-specific nitrosamines. While tar and nicotine are often highlighted regarding the harm of traditional tobacco, reports indicate that not only the particulate phase of tobacco, but also its gas phase has various effects on the body [4, 5]. Research indicates that the gas phase of tobacco activates nicotinamide adenine dinucleotide phosphate (NADPH) and induces cytotoxicity. Furthermore, the tobacco gas phase increases reactive oxygen species (ROS) in human umbilical vein endothelial cells (HUVECs), and inhibits thromboxane A2, thereby affecting platelet aggregation [6, 7]. In recent years, there has been a global surge in demand for heated tobacco products (HTPs). These products heat processed tobacco leaves at temperatures far below the combustion temperature of combustible cigarettes to generate nicotine-containing aerosols, which users inhale into their lungs. In the United States, brands of HTPs that are permitted for sale include IQOS (‘I-Quit-Ordinary-Smoking’; Philip Morris International [PMI]), Eclipse (R. J. Reynolds [RJR]) and Glo (British American Tobacco [BAT]), while Ploom (Japan Tobacco Inc [JTI]) is sold in Japan. The global HTPs market was valued at 13.6 billion USD in 2021 and is expected to grow to 947.8 billion USD by 2030 [8]. Although HTPs are purported to reduce the amounts of harmful and potentially harmful substances generated, further research is necessary to understand the short-term and long-term health impacts of HTPs.

The toxicity of traditional tobacco is known to be associated with the involvement of reactive oxygen ROS and Ca^2+^ [9, 10]. On the other hand, there is little knowledge of these in the context of HTPs. ROS act as second messengers and play a role in regulating normal physiological processes and cellular homeostasis [11]. To regulate ROS levels, various mechanisms exist, including localized generation, detoxification by antioxidant factors. In human cells, there are many enzymes, such as NAPDH oxidases, that generate hydrogen peroxide and superoxide [12]. However, when ROS are produced excessively, they can induce oxidative stress, resulting in damage to biomolecules and contributing to the development of various diseases, including those affecting the respiratory, neurodegenerative, and digestive systems. The production of ROS in smoking is well-known, and the presence of ROS in tobacco smoke is a significant factor in the oxidative stress and damage caused by smoking [9].

Ca^2+^ is also a versatile second messenger that is involved in multiple functions in eukaryotic cells, such as signal transduction, muscle contraction, apoptosis, cell cycle progression, cell migration and cell proliferation [13]. They function as a signal transduction pathway, in the release of neurotransmitters from neurons, in the contraction of all types of muscle cells, and as a second messenger in fertilization. Many enzymes, including several coagulation factors, require Ca^2+^ as a cofactor. However, high intracellular Ca^2+^ concentrations can become toxic, leading to cell death either through necrosis or apoptosis. This is often due to sustained high levels of Ca^2+^ in mitochondria, which can trigger the release of cytochrome c, followed by the activation of caspases and subsequent death signaling. While Ca^2+^ has important functions in the body, there are multiple reports that conventional tobacco affects intracellular calcium levels. When HL60 cells were stimulated with particulate phase of traditional tobaccos, an increase in intracellular Ca^2+^ was observed, which was not seen with nicotine alone [6]. Furthermore, it has been reported that whole particulate phase from traditional tobacco causes an increase in cytosolic Ca^2+^ concentration, and components of tobacco such as 1-NH2-naphthalene, formaldehyde, nicotine, and nicotine-derived nitrosamine ketone may be involved [10].

The manufacturers claim that HTPs have a reduced toxicity compared to traditional tobacco [14]. PMI reported that the levels of 40 out of the 93 harmful and potentially harmful constituents (HPHC) listed by the FDA are lower in IQOS aerosol than in traditional tobacco smoke [15]. On the other hand, an additional 56 other substances not included in PMI’s list or the FDA’s HPHC list were found at higher levels in IQOS emissions. Of these, 22 types were reported to be over 200% higher and 7 types were over 1000% higher compared to traditional tobacco smoke. Recently, it has become understood that heated tobacco also has toxicity. However, many aspects, such as the specific causes of this toxicity, remain poorly understood. Therefore, FDA does not recognize the reduction of health risks of heated tobacco. Due to differences in the chemicals produced, HTPs may elicit a different pharmacological action of cytotoxicity compared to traditional tobacco.

In the current study, we present our findings on the cytotoxic effects of nicotine- and tar-free cigarette smoke extract (CSE) from heat-not-burn tobacco products (HTPs) and traditional tobacco on oral squamous cell carcinoma (OSCC) cell lines (HSC-3, SCC-25 and OSC-19), as well as on some normal cell lines.

## Materials and methods

### CSE

The HTPs composing of a heating device (IQOS ILUMA, PMI) and tobacco sticks (TEREA Regular, PMI) was commercially obtained in Japan. Combustible reference cigarettes (1R6F) were purchased from the Center for Tobacco Reference Products at the University of Kentucky College of Agriculture (Lexington, KY, USA). The mainstream smoke from HTPs and traditional tobacco was generated according to the Cooperation Centre for Scientific Research Relative to Tobacco (CORESTA) technical report (HTP-259-CTR) smoking regime for high-temperature HTPs (55 mL puff volume, 30 s puff interval, 2 s puff duration, bell-shaped puff profile, and no blocking of the filter ventilation holes) and the Health Canada Intense (HCI) smoking regime (55 mL puff volume, 30 s puff interval, 2 s puff duration, bell-shaped puff profile, and 100% blocked filter ventilation holes), respectively, by using an analytical vaping machine (LM5E; Körber Technologies Instruments GmbH, Hamburg, Germany) [16, 17]. The particulate phase in the mainstream smoke was collected on a Cambridge filter pad (Körber Technologies Instruments GmbH). The resulting gas phase of ten cigarettes (eight puffs from one cigarette per trial) was bubbled into 5 mL of ice-cold, fetal bovine serum (FBS)-free cell culture media to prepare 80 puffs of CSE solution. The concentration of each CSE solution was considered 100%.

### Cell line

Human oral squamous cell carcinoma cell line HSC-3 was purchased from Health Science Research Resources Bank (Japan Health Sciences Foundation). Cell was cultured in High glucose Dulbecco’s modified Eagle’s medium (DMEM; Wako) supplemented with 10% FBS, and 5% penicillin/streptomycin (Thermo Scientific, IL, USA) in an incubator containing 5% CO_2_ at 37 °C. SCC-25 was obtained from American Type Culture Collection (USA). The OSC-19 used in the supplement data was obtained from the Health Science Research Resources Bank. Normal human astrocyte (NHA) was purchased from Lonza (Basel, Switzerland). Human gingival fibroblasts (HGnF) and oral keratinocytes were purchased from ScienCell Research Laboratories (Carlsbad, CA, USA) HUVEC was purchased from PromoCell (Heidelberg, Germany).

### Inhibitor

N-Acetylcysteine (NAC) was purchased from Wako (Osaka, Japan). In this experiment, NAC was used at a concentration of 1250 μM.

### Cell viability assay

Cell Counting Kit-8 (CCK-8; Dojin, Kumamoto, Japan) assay was used for cell proliferation viability. Briefly, cells were seeded in a 96-well plate at 5,000 cells per well and cultured for 24 h. In some experiments, we considered for a longer time (24, 48 and 72 h). We added 10 μl of reagent after stimulation, incubated for 2 h, and then measured the absorbance at 450 nm [18].

### Apoptosis assay

HSC-3 cells were incubated with or without CSE for 18 h. Flow cytometry using BD fluorescence-activated cell sorting (FACS) Canto II (BD Biosciences, CA, USA) were carried out as previously described. FITC Annexin V Apoptosis Detection Kit with 7-AAD (BioLegend, SD, USA) was used to stain the cells. Cells were divided into four sections. Q1, Q2, Q3, and Q4 marked in each panel indicate the necrosis, late apoptosis, early apoptosis, and lived cells, respectively.

### Western blotting

Western blot analyses were carried out as previously described. Briefly, cells were lysed and sonicated in RIPA buffer (Thermo Scientific, IL, USA). The following primary antibodies were used for immunoblotting: phosphor-p38, p38 obtained from CST (Cell Signaling Technology, Danvers, MA, USA), GAPDH obtained from Santa Cruz Biotechnology (Dallas, TX, USA). The calcium/calmodulin-dependent protein kinase kinase 2 (CaMKK2) antibody used to confirm the knockdown efficiency of CaMKK2 was purchased from CST (Supplementary Fig. 3). Chemiluminescence detection was performed using ECL reagent (Bio-Rad Laboratories, CA, USA) and high-sensitivity ECL reagent (Thermo Scientific). Signals were visualized using a LuminoGraph II imaging system (ATTO, Tokyo, Japan). The signal intensities of the bands were quantified using ATTO CS Analyzer 4 software (ATTO).

### ROS assay

To investigate the production of ROS, high-sensitive 2ʹ,7ʹ-dichlorofluorescein diacetate assay (HS-DCFH: Dojin) and Electron Spin Resonance (ESR) was performed.

#### HS-DCFH assay

Cells were incubated with Hanks’ Balanced Salt Solution (HBSS+; Wako) buffer containing of HS-DCFH working solution, followed by washing and incubation with HBSS+. After the CSE stimulation, measurements were performed using a microplate reader (Nivo, PerkinElmer, MA, USA). Fluorescence signals were measured with a microscopy system using an excitation wavelength of 485 nm and an emission of 535 nm.

#### ESR

ROS production was evaluated using an EMX-8/2.7 ESR spectrometer (Bruker Biospin, Billerica, MA, USA) with/without CSE. 5,5-dimethyl-pyrroline *N*-oxide (DMPO) was purchased from Labotec (Tokyo, Japan). The sample was injected into a capillary tube and inserted into an ESR measurement device and measured. Testing parameters were set as follows: 20 mW microwave power, 9.85 GHz microwave frequency, 30 s conversion time, five times scans, 2 G field modulation, and 100 G scan range.

### Fluorescence imaging of intracellular Ca^2+^

Cells were incubated with Hanks’ Balanced Salt Solution (HBSS+;Wako) buffer containing 4 µmol/l of Fluo-4AM (Dojin. Kumamoto, Japan), followed by washing and incubation with HEPES-buffered saline containing 2.0 mmol/l of CaCl_2_. An Eclipse Ti (Nikon Corporation, Tokyo, Japan) was used to monitor fluorescence changes. Full images were collected every 5 s. Fluo-4 fluorescence was excited at 488 nm, and data were expressed as normalized changes in background-corrected fluorescence emission (F/F0). Fluorescence signals were measured at room temperature using an excitation wavelength of 488 nm and an emission of 522.5 nm. Representative Ca^2+^ signals averaged from 50 individual cells were analyzed.

### Short-hairpin RNA transduction

HSC-3 cells were subjected to transduction with CaMKK2 shRNA and scramble control shRNA. Lentiviral transduction by VectorBuilder (Chicago, USA), Sigma‒Aldrich (MO, USA) and SCBT (Santa Cruz Biotechnology, CA, USA) was performed as previously described [19]

The transfection efficiency of the shRNAs was evaluated by Western blotting. Transductions with lentivirus were carried out as previously described. The sequences are shown in Supplementary Table 1.

### Data analysis and statistics

Statistical analysis was performed using GraphPad Prism 9 software (GraphPad Software Inc., San Diego, CA, USA). Statistical comparisons between two groups were performed using Student’s t test. Comparisons among more than two groups were performed using one-way analysis of variance (ANOVA) followed by Tukey’s test or by two-way ANOVA followed by the Bonferroni post hoc test. The criterion for statistical significance was set at p < 0.05.

### Large language model (LLM)

ChatGPT (GPT-4) was used only for English proofreading.

## Results

### Substances in the CSE derived from both HTPs and traditional tobacco exhibited cytotoxicity in OSCC cells. Additionally, CSE derived from HTPs had the potential to induce cell proliferation.

**Fig. 1:**
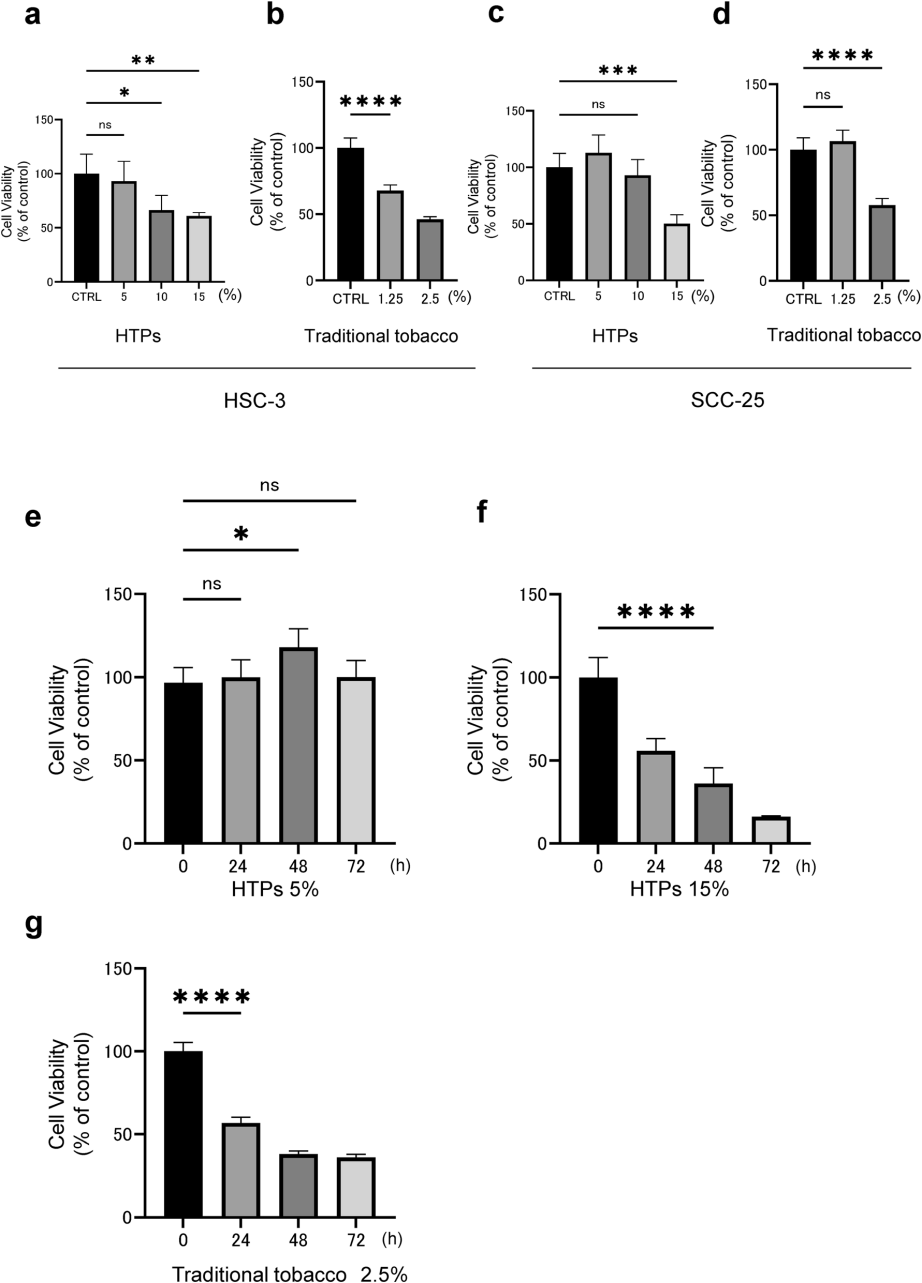
Substances derived from both HTPs and traditional tobacco products in the CSE exhibited cytotoxicity in HSC-3 cells. **a** Change in cell proliferation rate of HSC-3 cells after a 24-h exposure to various concentrations of CSE derived from HTPs. **b** Change in the cell proliferation rate of HSC-3 cells after a 24-h exposure to various concentrations of CSE derived from traditional tobacco. **c** Change in the cell proliferation rate of SCC-25 cells after a 24-h exposure to various concentrations of CSE derived from HTPs. **d** Change in the cell proliferation rate of SCC-25 cells after a 24-h exposure to various concentrations of CSE derived from traditional tobacco. **e** Long-term change in the cell proliferation rate after a 5% CSE derived from HTPs exposure. **f** Long-term change in the cell proliferation rate after a 15% CSE derived from HTPs exposure. **g** Long-term change in the cell proliferation rate after a 2.5% CSE derived from traditional tobacco exposure. Statistics: one-way ANOVA (and nonparametric), *n* = 4, ns; not significant, *; *p* < 0.05, **; *p* < 0.01, ***; *p* < 0.001, ****; *p* < 0.0001

To evaluate the effects of substances in the CSE derived from HTPs and traditional tobacco, we conducted a CCK-8 assay in OSCC cells (HSC-3 and SCC-25). Substances in the CSE derived from HTPs suppressed cell viability in a dose-dependent manner (Fig. 1a and c). Similarly, substances in the CSE derived from traditional tobacco also suppressed cell viability in a dose-dependent manner (Fig. 1b and d). Furthermore, we confirmed that substances derived from both HTPs and traditional tobacco products in the CSE also exhibited cytotoxicity in a range of cell types including OSC-19, HGnF, HUVEC, and NHA (Supplementary Fig. 1, a–h).

Interestingly, the CSE from HTPs at low concentrations (5%) appeared to facilitate cell proliferation, while at high concentrations (15%) they demonstrated time-dependent cytotoxic effects (Fig. 1e and f). Furthermore, substances in the 2.5% CSE derived from traditional tobacco suppressed cell proliferation of HSC-3 in a time-dependent manner (Fig. 1g). Our results indicated that high concentrations of CSE from HTPs exhibit cytotoxicity similar to CSE from traditional tobacco, but low concentrations of CSE from HTPs have shown the potential to induce cell proliferation.

### Substances in the CSE derived from both HTPs and traditional tobacco promoted cell apoptosis and phosphorylated p38 in OSCC cells

**Fig. 2:**
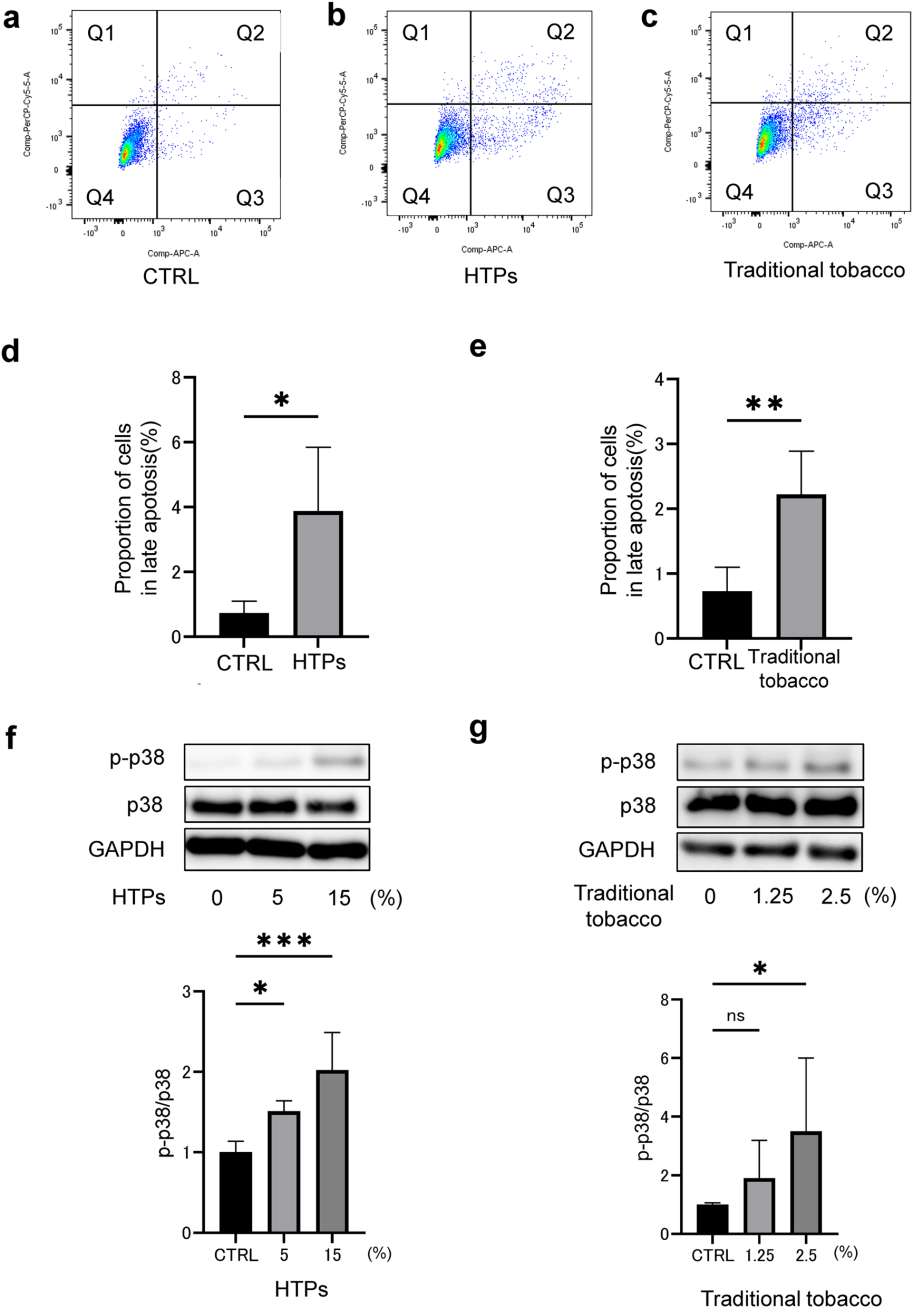
Substances derived from both HTPs and traditional tobacco in the CSE enhanced cytotoxicity and induced p38 phosphorylation. **a** Apoptosis assay in the control group. Q1, Q2, Q3, and Q4 marked in each panel indicate the necrosis, late apoptosis, early apoptosis, and lived cells, respectively. **b** Apoptosis in HSC3 cells 18 h after exposure to 15% CSE derived from HTPs exposure. **c** Apoptosis in HSC3 cells 18 h after exposure to 2.5% CSE derived from traditional tobacco exposure. **d** Late apoptosis (percentage of Q2) 18 h after exposure to 15% CSE derived from HTPs exposure. **e** Late apoptosis (percentage of Q2) 18 h after exposure to 2.5% CSE derived from traditional tobacco exposure. **f** Western blot (WB) analysis showing p38 phosphorylation following exposure to 15% CSE derived from HTPs. **g** WB analysis showing p38 phosphorylation following exposure to 2.5% CSE derived from traditional tobacco. Statistics: one-way ANOVA (and nonparametric), Tukey’s multiple comparisons test; **d** and **e** (*n* = 4), **f** (*n* = 5), **g** (*n* = 6), ns; not significant, *; *p* < 0.05, **; *p* < 0.01, ***; *p* < 0.001

In our subsequent research, we examined how tobacco affects cell apoptosis in OSCC cells. We found that CSE derived from HTPs, specifically IQOS, and traditional tobacco products, like 1R6F (the each amount that shows the same effect of each CSEs on cell viability), induced late-stage apoptosis in HSC-3 cells (Fig. 2a–e).

p38 protein belongs to the mitogen-activated protein kinases family that link extracellular stimuli with intracellular responses participating in a number of fundamental cell processes [20]. Gkouveris et al. have reported that the expression of p38 in OSCC cells may be involved in the proliferation of these cells through the mediation of signal transducer and activator of transcription 3.

Therefore, our investigation focused on this process to understand the underlying mechanism of tobacco-induced cytotoxicity in HSC-3 cells. Our findings confirmed that substances from CSE of both HTPs and traditional tobacco products indeed cause p38 phosphorylation in HSC-3 cells (as shown in Fig. 2f and g).

### Substances in the CSE derived from both HTPs and traditional tobacco increased ROS production in OSCC cells

**Fig. 3:**
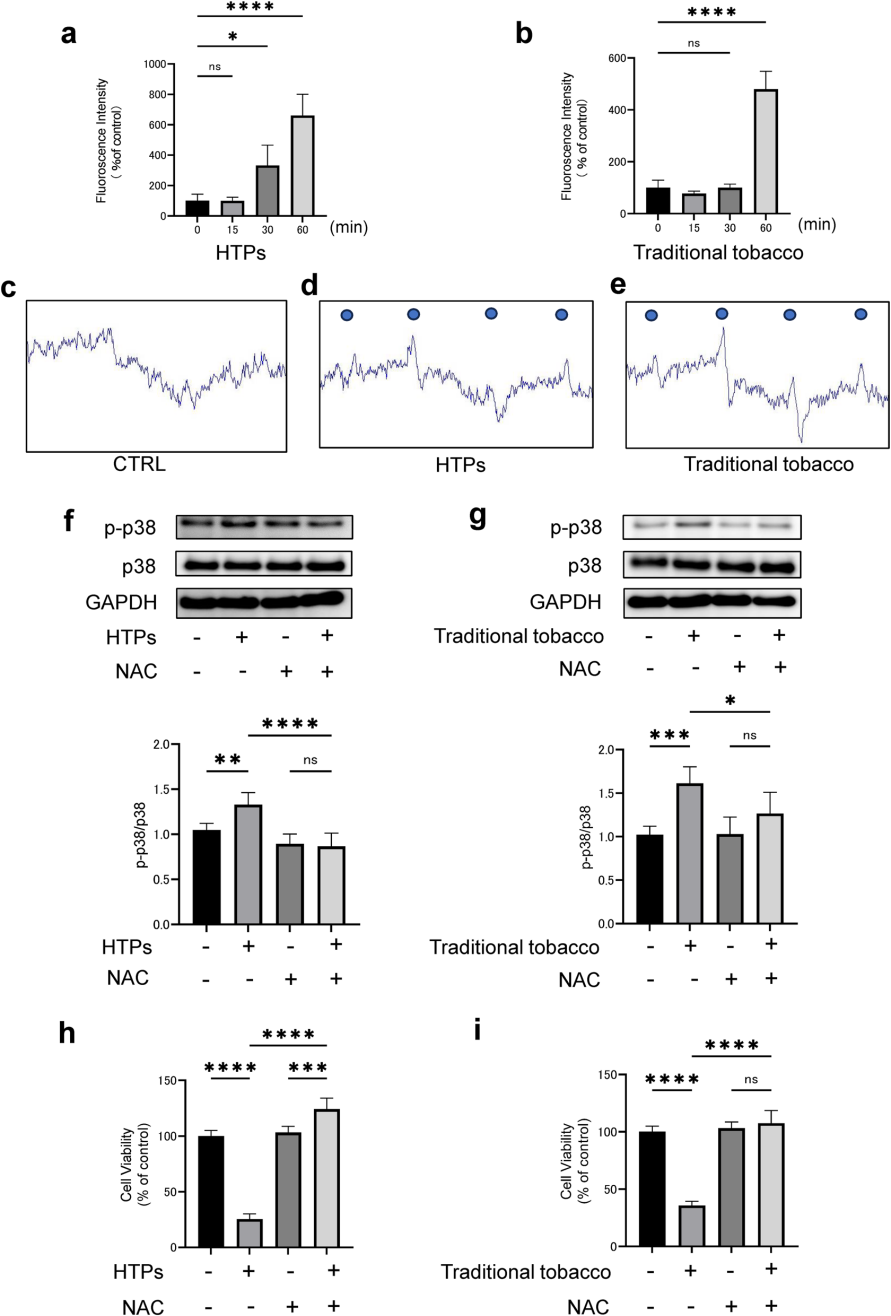
Substances derived from both HTPs and traditional tobacco in the CSE induced ROS production. **a** ROS measurement following exposure to 15% CSE derived from HTPs exposure, as determined by HS-DCFH assay. **b** ROS measurement following exposure to 2.5% CSE derived from traditional tobacco exposure, as determined by HS-DCFH assay. **c** Representative ESR result for ROS measurement in unstimulated cells. **d** Representative ESR result for ROS measurement following exposure to 15% CSE derived from HTPs. exposure. **e** Representative ESR result for ROS measurement following exposure to 2.5% CSE derived from traditional tobacco exposure. **f** WB analysis illustrating p38 phosphorylation following exposure to 15% CSE derived from HTPs in the presence of NAC (1250 μM). **g** WB analysis illustrating p38 phosphorylation following exposure to 2.5% CSE derived from traditional tobacco in the presence of NAC (1250 μM). **h** Cell proliferation under NAC (1250 μM) treatment24 h after exposure to 15% CSE derived from HTPs exposure. **i** Cell proliferation under NAC (1250 μM) treatment 24 h after exposure to 2.5% CSE derived from traditional tobacco exposure. Statistics: one-way ANOVA (and nonparametric), ns; not significant, *; *p* < 0.05, **; *p* < 0.01, ***; *p* < 0.001, ****; *p* < 0.0001, a and b (*n* = 4), **f** and **g** (*n* = 5), **h** and **i** (*n* = 6)

Our study found that substances from both HTPs and traditional tobacco products increased the production of ROS in OSCC cells. This was determined by measuring the HS-DCFH assay one hour after stimulation (Fig. 3a, b). When measured for one hour with CSE of HTPs or traditional tobacco at the same time, the ROS production was higher in 2.5% CSE of traditional tobacco than 15% CSE of HTPs (Supplemental Fig. 2). ESR spectroscopy, a highly regarded method for such analysis, indicated an increase in ROS production one hour post-stimulation with both types of tobacco. ESR spectroscopy exhibits characteristic four-line spectra when free radicals bind to DMPO (Fig. 3c–e).

It is well-established that ROS production induces apoptosis in cells via the p38 pathway [21]. Given these results showing increased ROS production by tobacco substances, we next evaluated the impact of NAC, a known antioxidant, on tobacco-induced cytotoxicity and p38 phosphorylation. Our findings revealed that NAC reduced the p38 phosphorylation triggered by both tobacco substances (Fig. 3f and g). Furthermore, NAC mitigated the cytotoxic effects induced by these tobacco substances (Fig. 3h and i). Interestingly, HSC-3 stimulated with CSE of HTPs combined with NAC led to cell proliferation compared to the control.

### Substances in the CSE derived from both HTPs and traditional tobacco increased intracellular Ca^2+^ concentration in OSCC cells

**Fig. 4:**
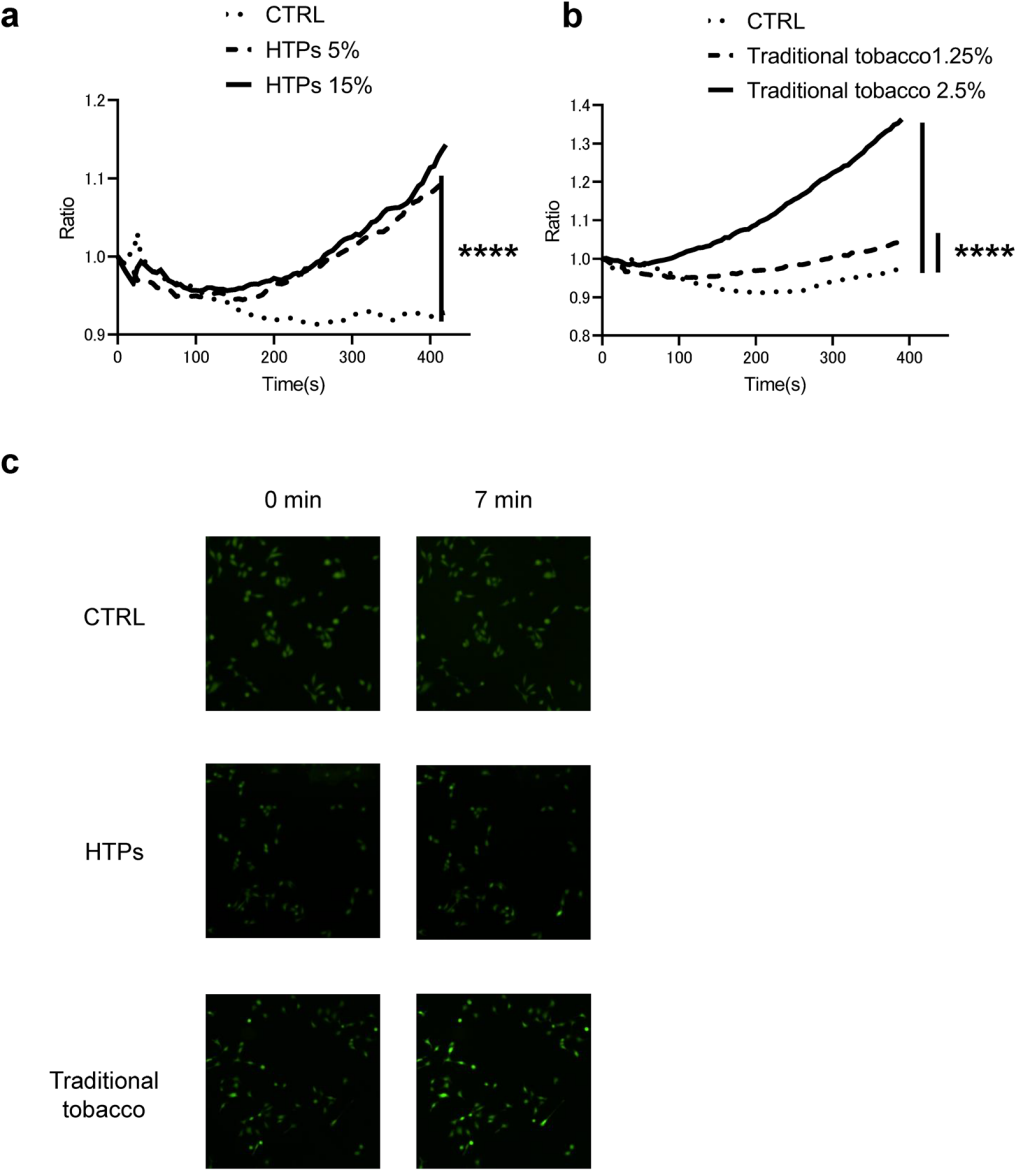
Substances derived from both HTPs and traditional tobacco in the CSE increased intracellular Ca^2+^ concentration. **a** Changes in intracellular Ca^2+^ concentration following stimulation with either 5% or 15% CSE derived from HTPs exposure. **b** Changes in intracellular Ca^2+^ concentration following stimulation with either 1.25% or2.5% CSE derived from traditional tobacco exposure. **c** Representative fluorescence image of Ca^2+^ concentration before and 7 min after exposure to CSE derived from either HTPs or traditional tobacco. Statistics: one-way ANOVA (and nonparametric), ****; *p* < 0.0001, 50 cells were randomly selected for analysis from the microscope images

Intracellular Ca^2+^, a ubiquitous second messenger, is vital in regulating numerous cellular processes, such as proliferation, apoptosis and cell migration, including in cancer cells [13]. Wylam et al. previously reported that substances derived from traditional tobacco products increased intracellular Ca^2+^ concentration [22]. To observe the effect of substances derived from both HTPs and traditional tobacco products on intracellular Ca^2+^ concentration, we measured the intracellular Ca^2+^ levels using the Fluo-4 assay, a Ca^2+^-sensitive dye, immediately after stimulation with these tobacco product ups to 7 min post-stimulation. Similarly, our study found that substances in the CSE derived from both HTPs and traditional tobacco products also elevated intracellular Ca^2+^ concentration in HSC-3 cells as well (Fig. 4a–c).

### Knockdown of CaMKK2 attenuated the CSE derived from HTPs and traditional tobacco substance-induced cytotoxicity, p38 phosphorylation and ROS production

**Fig. 5:**
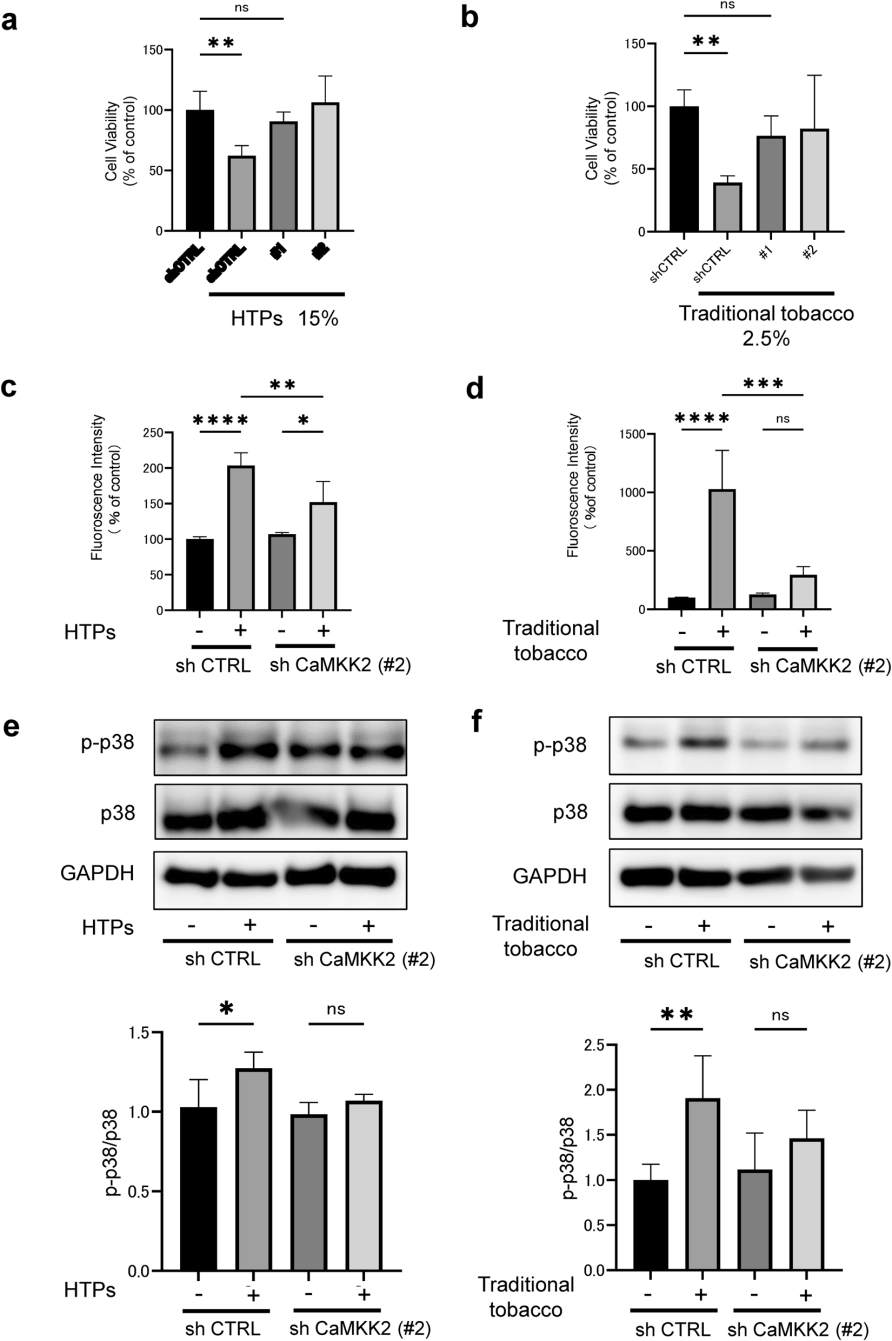
Knockdown of CaMKK2 mitigates cytotoxicity, ROS production and p38 phosphorylation in HSC3 cells induced by substances derived from both HTPs and traditional tobacco in the CSE. **a** Change in cell proliferation rate in CaMKK2 knockdown HSC-3 cells (#1 and #2) following a 24-h exposure to 15% CSE derived from HTPs. **b** Change in cell proliferation rate in CaMKK2 knockdown HSC-3 cells (#1 and #2) following a 24-h exposure to 2.5% CSE derived from traditional tobacco. **c** Measurement of ROS in CaMKK2 knockdown HSC-3 cells (#2) following exposure to 15% CSE derived from HTPs exposure, as determined by the HS-DCFH assay. **d** Measurement of ROS in CaMKK2 knockdown HSC-3 cells (#2) following exposure to 2.5% CSE derived from traditional tobacco exposure, as determined by the HS-DCFH assay. **e** WB analysis illustrating p38 phosphorylation in CaMKK2 knockdown HSC-3 cells (#2) following exposure to 15% CSE derived from HTPs. **f** WB analysis illustrating p38 phosphorylation in CaMKK2 knockdown HSC-3 cells (#2) following exposure to 2.5% CSE derived from traditional tobacco. Statistics: one-way ANOVA (and nonparametric), *n* = 4, ns; not significant, *; *p* < 0.05, **; *p* < 0.01, ***; *p* < 0.001

Following our finding that tobacco substances quickly elevate intracellular Ca^2+^ levels, we shifted our focus to CaMKK2, a calcium-associated protein. Initially, we developed two types of HSC-3 cells with CaMKK2 knockdown (#1 and #2) using shRNA lentivirus and assessed the effects on cytotoxicity, ROS production, and p38 phosphorylation induced by these tobacco substances. We conducted western blotting to investigate the efficiency of CaMKK2 knockdown. It was confirmed that CaMKK2 was significantly suppressed in comparison to the control shRNA (Supplemental Fig. 4). Our results showed that CaMKK2 knockdown reduced cytotoxicity in HSC-3 cells caused by tobacco substances (Fig. 5a and b). Subsequent experiments only used #2, which showed more pronounced suppression of CaMKK2, for comparison with Sh-CTRL. Additionally, CaMKK2 knockdown cells decreased ROS production induced by both tobacco substances (Fig. 5c and d). Furthermore, CaMKK2 knockdown cells also mitigated p38 phosphorylation triggered by both types of tobacco substances (Fig. 5e and f).

## Discussion

**Fig. 6:**
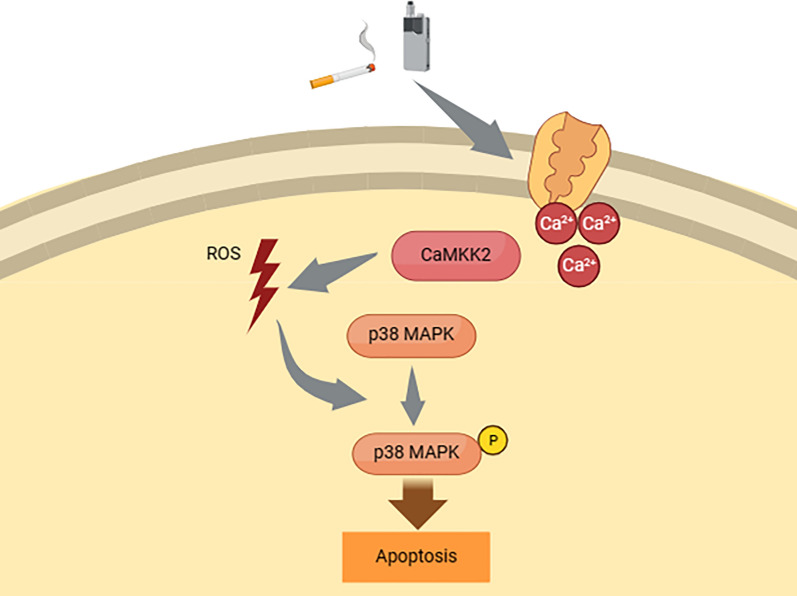
Hypothetical pathway of apoptosis stimulation by CSE derived from both HTPs and traditional tobacco. The findings indicate that, similar to traditional tobacco, HTPs induce apoptosis via ROS/p38 signaling pathways. These pathways could potentially be regulated by CaMKK2

In this research, we compared and verified the known mechanisms of toxicity in traditional tobacco with those in HTPs and investigated whether there are any unidentified signaling pathways. Our study is the first to reveal that the substances in the CSE derived from both HTPs and traditional tobacco promoted cell apoptosis and suppressed cell proliferation through the ROS/p38 pathway and CaMKK2 in OSCC cells. This experiment’s findings are presented in a schematic diagram (Fig. 6). Additionally, CSE derived from HTPs, at certain concentrations, induced cell proliferation, during this process enhanced phosphorylation of p38 and increased intracellular Ca^2+^ concentration occurred.

Several reports indicate that extracts from the CSE derived from IQOS exhibit lower cytotoxicity than traditional tobacco [23–25]. In addition, there are a few reports that heated tobacco caused cell proliferation, and there is also a report that CSE contributed to proliferation [26–28]. However, the mechanisms of cytotoxicity and cell proliferation brought about by HTPs were not yet fully understood.

In our study, whether at concentrations that induce cell proliferation or those that cause apoptosis, phosphorylation of p38 was enhanced with CSE derived from HTPs. Phosphorylation of p38 can induce apoptosis through various downstream signaling pathways [21]. Additionally, multiple studies have reported that p38 may be involved in cell proliferation [20, 29, 30]. There are several reports suggesting that traditional tobaccos cause phosphorylation of p38, which is implicated in carcinogenesis and toxicity [31, 32]. Furthermore, the apoptosis mechanism in traditional tobacco is reported to involve the ROS/p38 pathway [33]. Although it has been previously reported that IQOS aerosols can enhance p38 in rat liver cells, our findings suggest that this effect might be attributed to the gas phase of the aerosol [34]. There are also reports that the CSE of HTPs has overactivated p53 [35].

Previous reports have acknowledged that the toxicity of traditional tobacco involves the generation of ROS and an increase in intracellular Ca^2+^ concentration [4, 22, 36, 37]. Similarly, in our study, CSEs derived from HTPs and traditional tobacco were found to increase ROS activity and intracellular Ca^2+^ concentration in OSCC cells. For both types of tobacco, suppressing ROS production also reduced cytotoxicity and inhibited the phosphorylation of p38. Notably, combining CSE from HTPs with NAC resulted in cell proliferation. Since combining CSE from HTPs with NAC also resulted in the suppression of phosphorylation of p38, there is a possibility that cell proliferation was induced by other mechanisms, such as other Ca^2+^-related signals.

It is reported that in traditional tobacco, stimulating human pulmonary artery smooth muscle cells (PASMCs) and endothelial cells (PAECs) leads to COX-2 production, which in turn drives cell proliferation [38]. This cell proliferation is considered abnormal remodeling, suggesting that excessive cell growth can lead to damage in lung tissue. There are multiple reports suggesting that the gaseous components of cigarette smoke can accelerate the cell cycle, leading to uncontrolled cell proliferation, which ultimately may result in carcinogenesis [39]. Similarly, it has been reported that in the case of IQOS, stimulation can lead to accelerated aging of vascular smooth muscle cells [40].

In our study, we observed an increase in intracellular Ca^2+^ concentration in OSCC cells. To evaluate the toxicity of tobacco extracts on cells, we knocked down CaMKK2, which is associated with Ca^2+^ elevation, using lentivirus. The results showed that knockdown of CaMKK2 reduced cytotoxicity and decreased ROS production in cells exposed to both HTPs and traditional tobacco. These findings suggest that CaMKK2 may play a role upstream of ROS production.

In this study, the equivalent of 80 puffs of IQOS was extracted into 5 ml of medium. Other literature adjusts IQOS to about 25 puffs/5 ml [24], and 2.5 puffs/5 ml [27], indicating that our study used a more concentrated condition for creating CSEs. For traditional tobacco, a 2.5% concentration involves dissolving the equivalent of 0.4 puffs in 1 ml of solution, whereas for HTPs (IQOS), a 15% concentration involves dissolving 2.4 puffs in 1 ml of solution. These smoking frequencies do not greatly deviate from the actual smoking frequencies of humans. However, it is challenging to directly correlate these CSE concentrations with in vivo conditions. Further research is required to understand how much of the gas-phase components of HTPs and traditional tobacco are actually absorbed and metabolized by body fluids and cells. While HTPs are marketed as safer, it can only be said that if the number of puffs is high, they may have cytotoxicity similar to traditional tobacco. Other in vitro experiments, like those spraying gas directly into the medium instead of using extracts [25], also exist, and differences in stimulation methods on cells must be considered.

Previous reports have acknowledged that the toxicity of traditional tobacco involves the generation of ROS and an increase in intracellular Ca^2+^ concentration [4, 22, 36, 37]. Similarly, in our study, CSEs derived from HTPs and traditional tobacco were found to increase ROS activity and intracellular Ca^2+^ concentration in OSCC cells. For both types of tobacco, suppressing ROS production also reduced cytotoxicity and inhibited the phosphorylation of p38. Notably, combining CSE from HTPs with NAC resulted in cell proliferation. Since combining CSE from HTPs with NAC also resulted in the suppression of phosphorylation of p38, there is a possibility that cell proliferation was induced by other mechanisms, such as other Ca^2+^-related signals.

A limitation of our study is that we only used the CSE from HTPs and traditional tobacco, without analyzing the individual substances in the extracts. Although we standardized the mass of each extract, the specific chemicals in them remain unidentified. While a comparison of toxicity is necessary, the different concentration conditions of HTPs and traditional tobacco mean that the efficacy of directly comparing changes in cellular signaling or ROS production is not necessarily guaranteed. In this study, we did not conduct in vivo experiments due to the challenges associated with testing how the extracts affect tumor cells in a living organism. However, it seems necessary for future research to examine the effects of extracts from HTPs and traditional tobacco on the body, especially on cancer cells.

In our study, we found that HTPs, like traditional tobacco, possess cytotoxicity, but their toxicity might be reduced as claimed by the manufacturers. The introduction of HTPs is touted as a 'harm reduction' strategy for individuals unable to quit nicotine addiction. However, there are reports suggesting that this strategy may be a rationale to circumvent regulations imposed on traditional tobacco products [41].

Substances like acrolein and formaldehyde, which generate ROS and cause toxicity, may be reduced in heated tobacco. However, there might be entirely different substances in heated tobacco that could promote cancer cell proliferation. While traditional tobacco, due to combustion, might produce a wider range of substances, heated tobacco could potentially control the chemicals generated by maintaining a constant temperature. Instead of simply considering heated tobacco as a lower-risk alternative as suggested by manufacturers, further research is needed focusing on the nature and characteristics of individual chemicals produced when using heated tobacco.

## Conclusion

Our findings suggest that the CSE from both HTPs and traditional tobacco induce cytotoxicity. This toxicity is mediated by ROS, which are regulated through Ca2+ signaling and CaMKK2 pathways. On one hand, CSE derived from HTPs causes cell proliferation at low concentrations, and identifying its mechanism remains a challenge for future research.

### Supplementary Information

Supplementary Material 1.

## Data Availability

The datasets used and/or analyzed during the current study are available from the corresponding author upon reasonable request.
