# Supplementary material for: Cytotoxic effects of the cigarette smoke extract of heated tobacco products on human oral squamous cell carcinoma: the role of reactive oxygen species and CaMKK2
Source: J Physiol Sci. 2024 Jun 25;74:35. doi: 10.1186/s12576-024-00928-1 (PMC11197199; doi:10.1186/s12576-024-00928-1)
Supplement: Supplementary file 1 — Supplementary Material 1. [file 12576_2024_928_MOESM1_ESM.docx]

| **Table S1: Short-heparin RNA transduction** | | |
| --- | --- | --- |
| **Species** | **ShRNA** | **Targeting sequences** |
| Human | Sh Control | CCTAAGGTTAAGTCGCCCTCG |
| Human | Sh CaMKK2-1(#1) | GTGAAGACCATGATACGTAAA |
| Human | Sh CaMKK2-2(#2) | CCCGATGCTTCTGTTTCATTC |

**Supplementary Table S1**

This table lists the targeting sequences used for short hairpin RNA (shRNA) transduction.


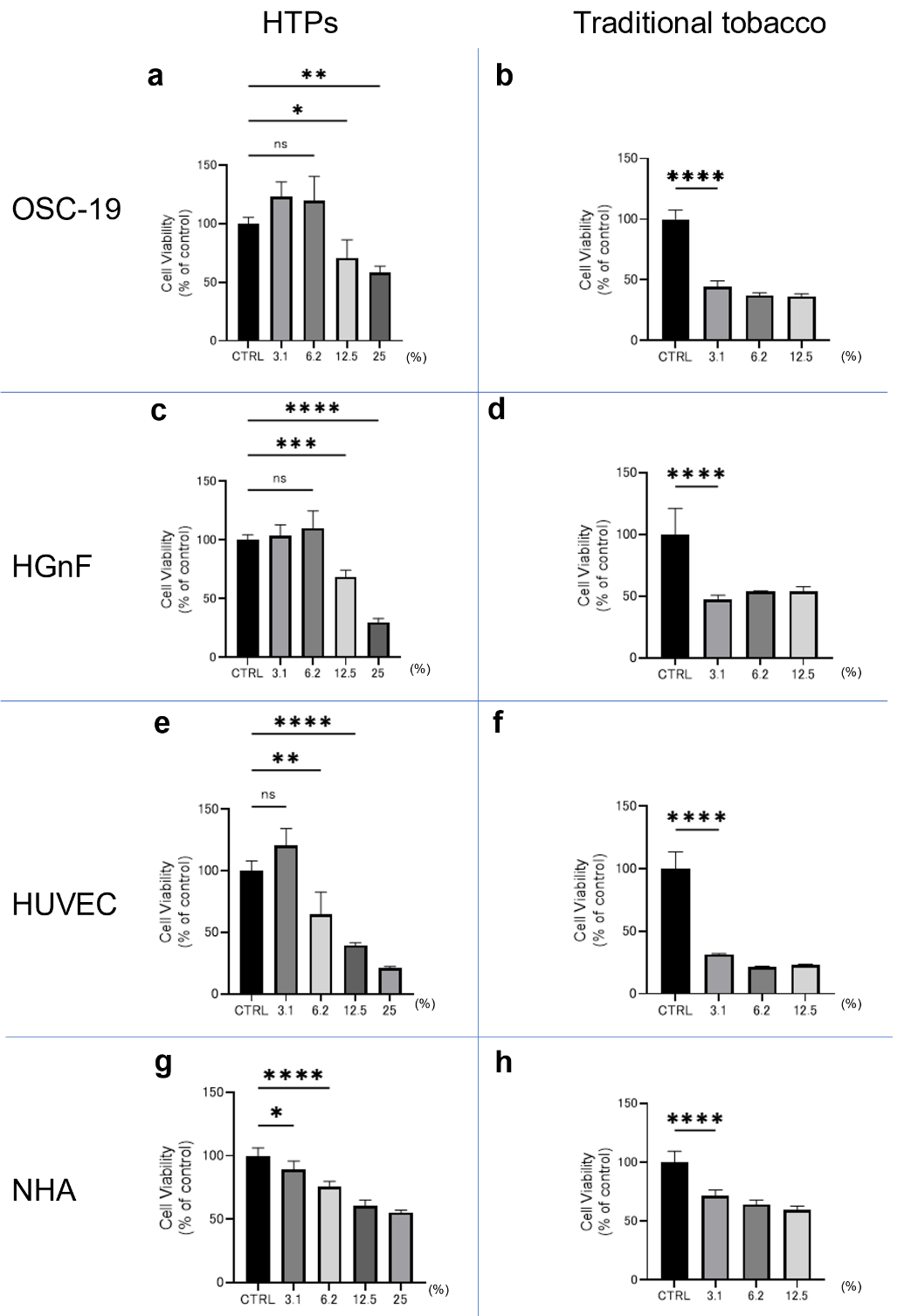


**Supplemental information1: The cytotoxic effects of substances in the cigarette smoke extract (CSE) derived from Heat-not-Burn Tobacco Products (HTPs) and traditional tobacco (1R6F) on various cell lines.**

It shows the changes in cell proliferation rates after 24 hours of stimulation with CSE from both sources. The cell lines tested include: a. OSC-19 (HTPs), b. OSC-19 (1R6F), c. Human Gingival Normal Fibroblasts (HGnF) (HTPs), d. HGnF (1R6F), e. Human Umbilical Vein Endothelial Cells (HUVEC) (HTPs), f. HUVEC (1R6F), g. Normal Human Astrocytes (NHA) (HTPs), h. NHA (1R6F).


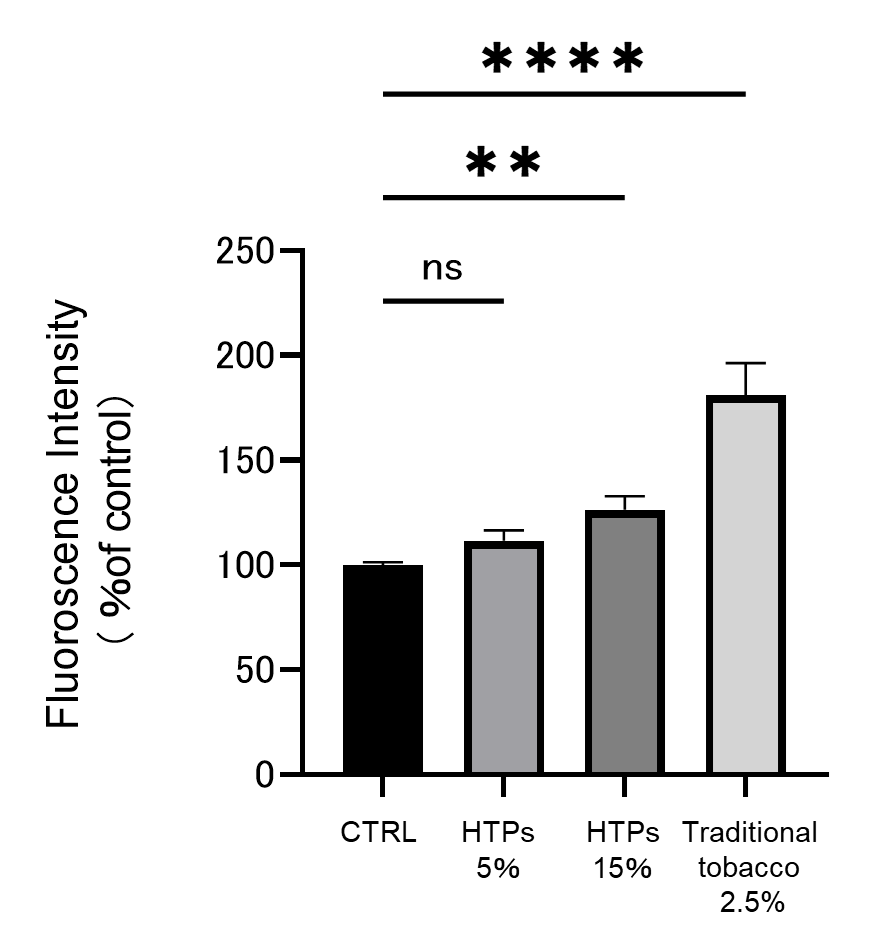


**Supplemental information 2:** **The measurement of Reactive Oxygen Species (ROS) following exposure to 5% or 15% IQOS (I Quit Ordinary Smoking) and 2.5% 1R6F.**

ROS levels were determined using the Hydrogen Peroxide Sensitive 2',7'-Dichlorofluorescin Diacetate (HS-DCFH) assay one hour after stimulation. ROS measurement following 5% or 15% IQOS of 2.5% 1R6F exposure, as determined by HS-DCFH assay one hours after stimulation. Statistics: One-way ANOVA (and nonparametric), n=4, ns; not significant, **; *p*<0.01, ****; *p*<0.0001.


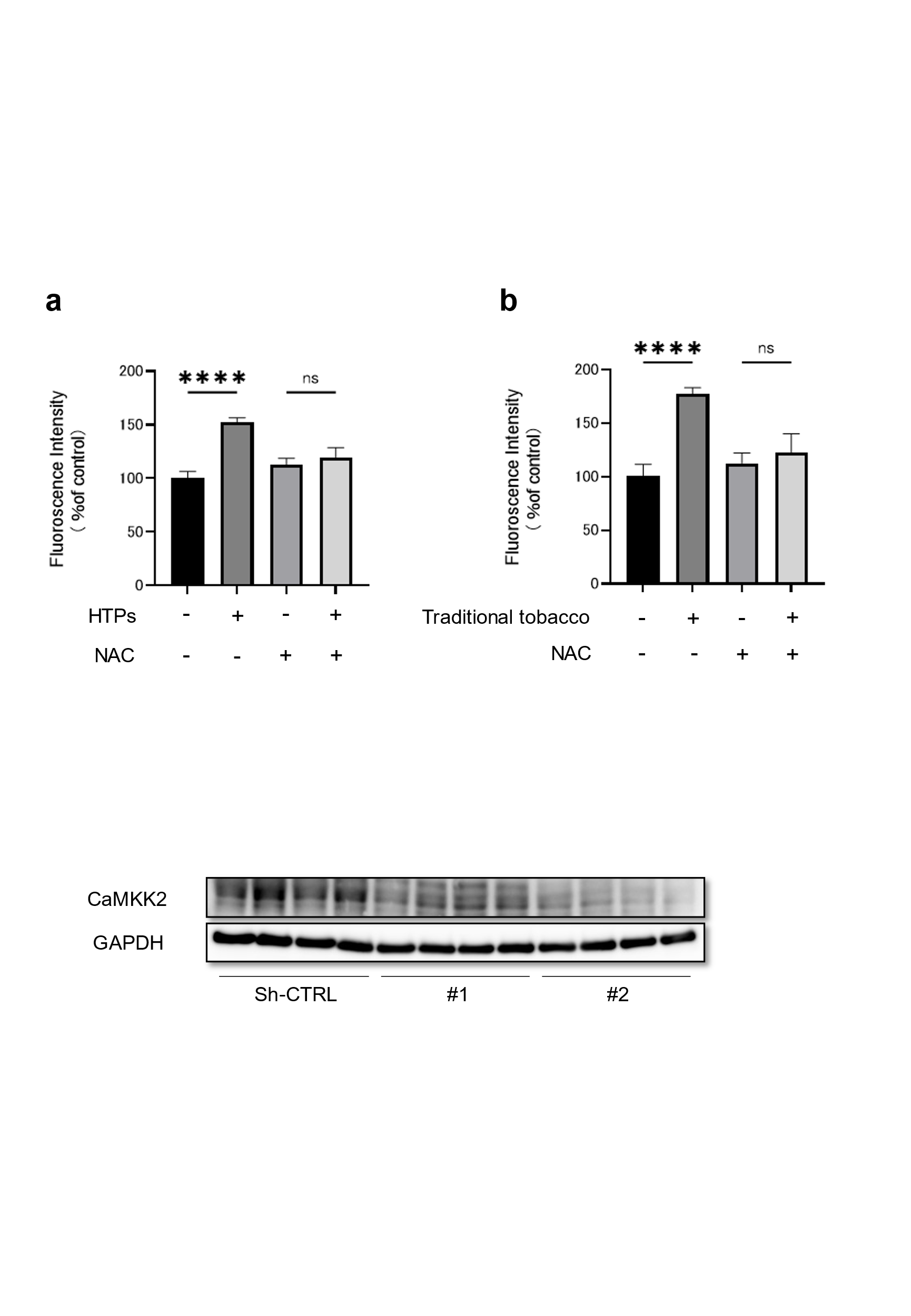


**Supplemental information 3: The measurement of ROS following exposure to 15% IQOS and 2.5% 1R6F in the presence of NAC.**

a. ROS measurement following exposure to 15% CSE derived from HTPs exposure, determined using the HS-DCFH assay in the presence of NAC (1250 μM).

b. ROS measurement following exposure to 2.5% CSE derived from traditional tobacco, determined using the HS-DCFH assay in the presence of NAC (1250 μM). Statistics: One-way ANOVA (and nonparametric), n=4, ns; not significant, ****; *p*<0.0001.

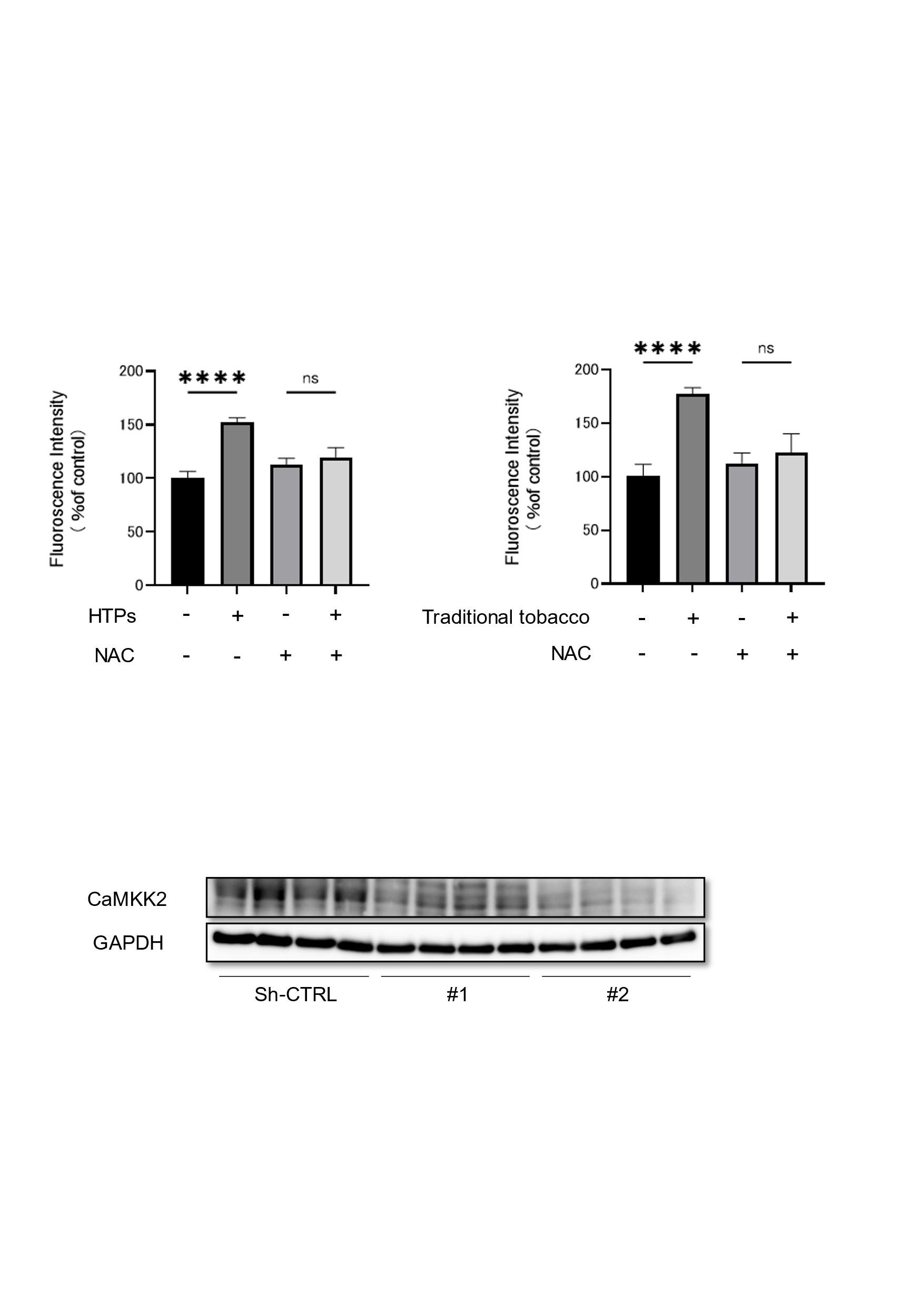


**Supplemental information 4: This figure presents the confirmation of CaMKK2 knockdown efficiency in HSC-3 cells, as evidenced by western blot analysis.**
